# Sepsis-induced myocardial dysfunction: from mechanisms to precision critical care

**DOI:** 10.1186/s40635-026-00957-7

**Published:** 2026-07-27

**Authors:** Sabri Soussi, Michelle S Chew

**Affiliations:** 1 Department of Anesthesiology and Pain Medicine, University Health Network, University of Toronto, Toronto, ON Canada; 2 University of Paris Cité, Inserm UMR-S 942, Cardiovascular Markers in Stress Conditions (MASCOT), Paris, France; 3 Department of Perioperative Medicine and Intensive Care, Division of Anesthesiology and Intensive Care, Karolinska University Hospital, CLINTEC, Karolinska Institutet, Stockholm, Sweden

Sepsis-induced myocardial dysfunction (SIMD) has emerged as one of the most challenging and least understood manifestations of sepsis. Although frequently contributing to circulatory failure and poor outcomes, its definition, pathophysiology, diagnosis, and therapeutic implications remain incompletely resolved. SIMD is characterized by marked heterogeneity in clinical presentation, temporal evolution, and reversibility, hindering the development of standardized diagnostic criteria, limiting comparisons across studies, and slowing progress toward targeted therapies [1].

Advances in cardiovascular imaging, biomarker research, and translational science have substantially improved our understanding of the interaction between the heart and the dysregulated host response during sepsis [1]. Increasing evidence suggests that SIMD is not a single disease entity but a syndrome comprising multiple biological and hemodynamic subphenotypes with distinct prognostic and therapeutic implications [2].

A major challenge remains the absence of a universally accepted definition. Historically, SIMD has been defined by reversible left ventricular systolic dysfunction assessed using left ventricular ejection fraction (LVEF), although LVEF is highly dependent on loading conditions and may not reflect intrinsic myocardial contractility in sepsis [3]. Reversibility is also poorly documented because most studies provide only a single early assessment and are limited by the competing risk of death. Contemporary approaches increasingly incorporate right ventricular function, diastolic dysfunction, myocardial deformation imaging, and ventricular–arterial coupling, yet uncertainty persists regarding which parameters best identify clinically meaningful dysfunction [3, 4].

Similarly, although cardiac troponins and natriuretic peptides are frequently elevated during sepsis, they lack specificity for SIMD [1, 3]. Emerging biomarkers of myocardial fibrosis, endothelial injury, glycocalyx degradation, mitochondrial dysfunction, inflammation, and metabolic adaptation may improve biological subphenotyping but require validation before routine clinical application [5].

Recent advances in precision medicine, artificial intelligence, and machine learning offer new opportunities to redefine SIMD beyond conventional clinical classifications. Integrating echocardiography, advanced hemodynamic monitoring and omics-based biomarkers may identify reproducible cardiovascular subphenotypes, improve prognostic stratification, and facilitate enriched clinical trials [6, 7].

This thematic collection, *Sepsis-induced myocardial dysfunction: from mechanisms to precision critical care*, invites original clinical and translational investigations that advance our understanding of myocardial dysfunction during sepsis. We welcome submissions addressing pathophysiological mechanisms, cardiovascular phenotyping, advanced imaging, biomarker discovery, advanced hemodynamic monitoring, right ventricular dysfunction, microcirculatory and mitochondrial dysfunction, artificial intelligence, novel therapeutic approaches, and mechanisms and phenotypes of long-term cardiovascular recovery after sepsis. By bringing together complementary research across these areas, this collection aims to advance the scientific basis for precision cardiovascular care in patients with SIMD.

## Data Availability

Not applicable.
